# Supplementary material for: Cataleptogenic Effect of Haloperidol Formulated in Water-Soluble Calixarene-Based Nanoparticles
Source: Pharmaceutics. 2023 Mar 11;15(3):921. doi: 10.3390/pharmaceutics15030921 (PMC10059056; doi:10.3390/pharmaceutics15030921)
Supplement: Supplementary file 1 [file pharmaceutics-15-00921-s001.zip › pharmaceutics-2261943-supplementary.pdf]

pharmaceutics

MDPI

Supplementary Materials

Cataleptogenic Effect of Haloperidol Formulated in Water-Soluble Calixarene-Based Nanoparticles

Nadezda E. Kashapova [1,*], Ruslan R. Kashapov [1,*], Albina Y. Ziganshina [1], Dmitry O. Nikitin [2], Irina I. Semina [2], Vadim V. Salnikov [3], Vitaliy V. Khutoryanskiy [4], Rouslan I. Moustafine [5] and Lucia Y. Zakharova [1]

1 Arbuzov Institute of Organic and Physical Chemistry, FRC Kazan Scientific Center of RAS, 8 Arbuzov Str., 420088 Kazan, Russia
2 Department of Pharmacology, Kazan State Medical University, 49 Butlerov Str., 420012 Kazan, Russia
3 Kazan Institute of Biochemistry and Biophysics, FRC Kazan Scientific Center of RAS, 2/31 Lobachevsky Str., 420111 Kazan, Russia
4 School of Pharmacy, University of Reading, Whiteknights, Reading, RG66DX, United Kingdom
5 Institute of Pharmacy, Kazan State Medical University, 16 Fatykh Amirkhan Str., 420126 Kazan, Russia
* Correspondence: kashapov@iopc.ru (R.R.K); kashapova.nadya@gmail.com (N.E.K)

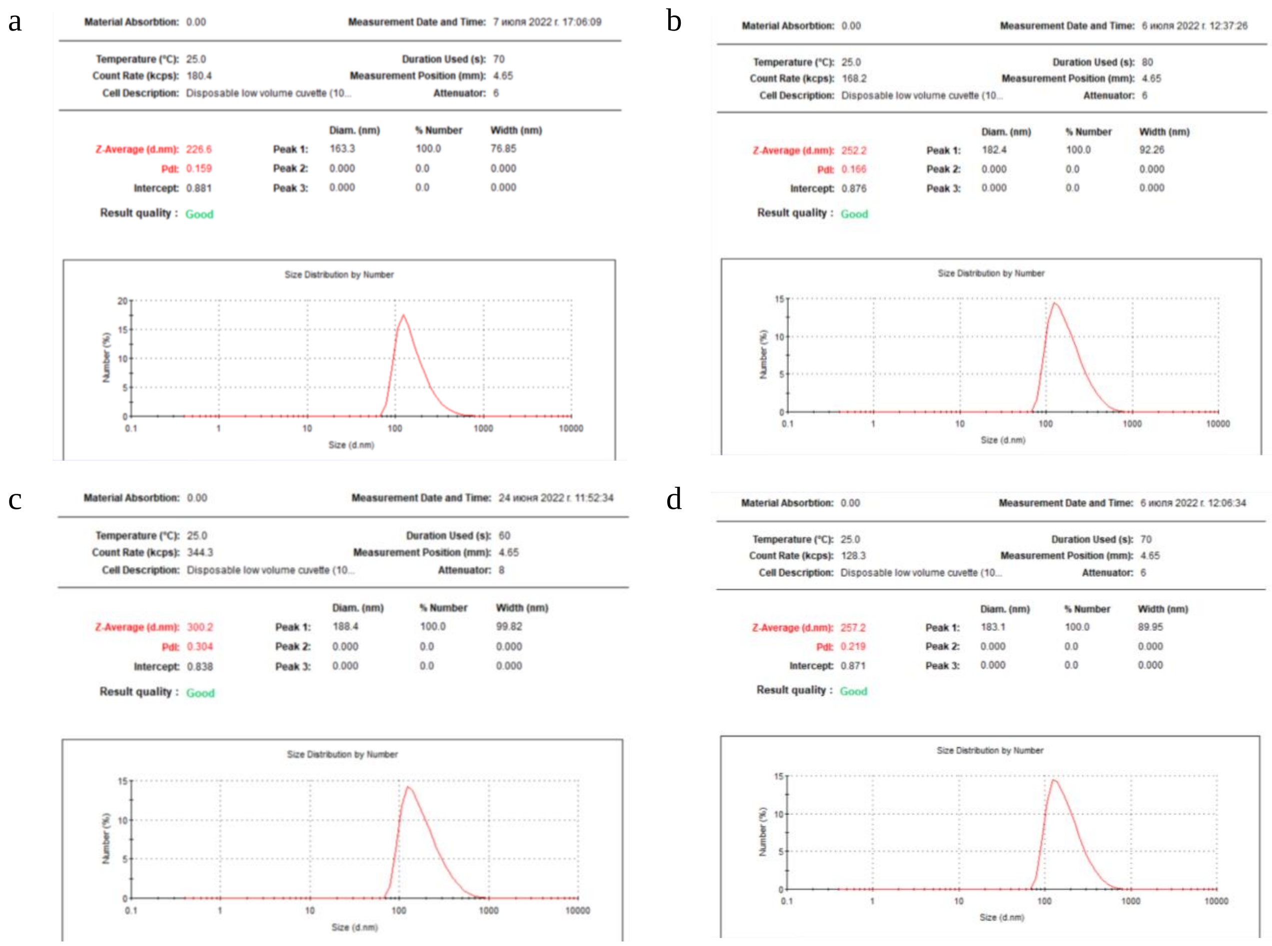

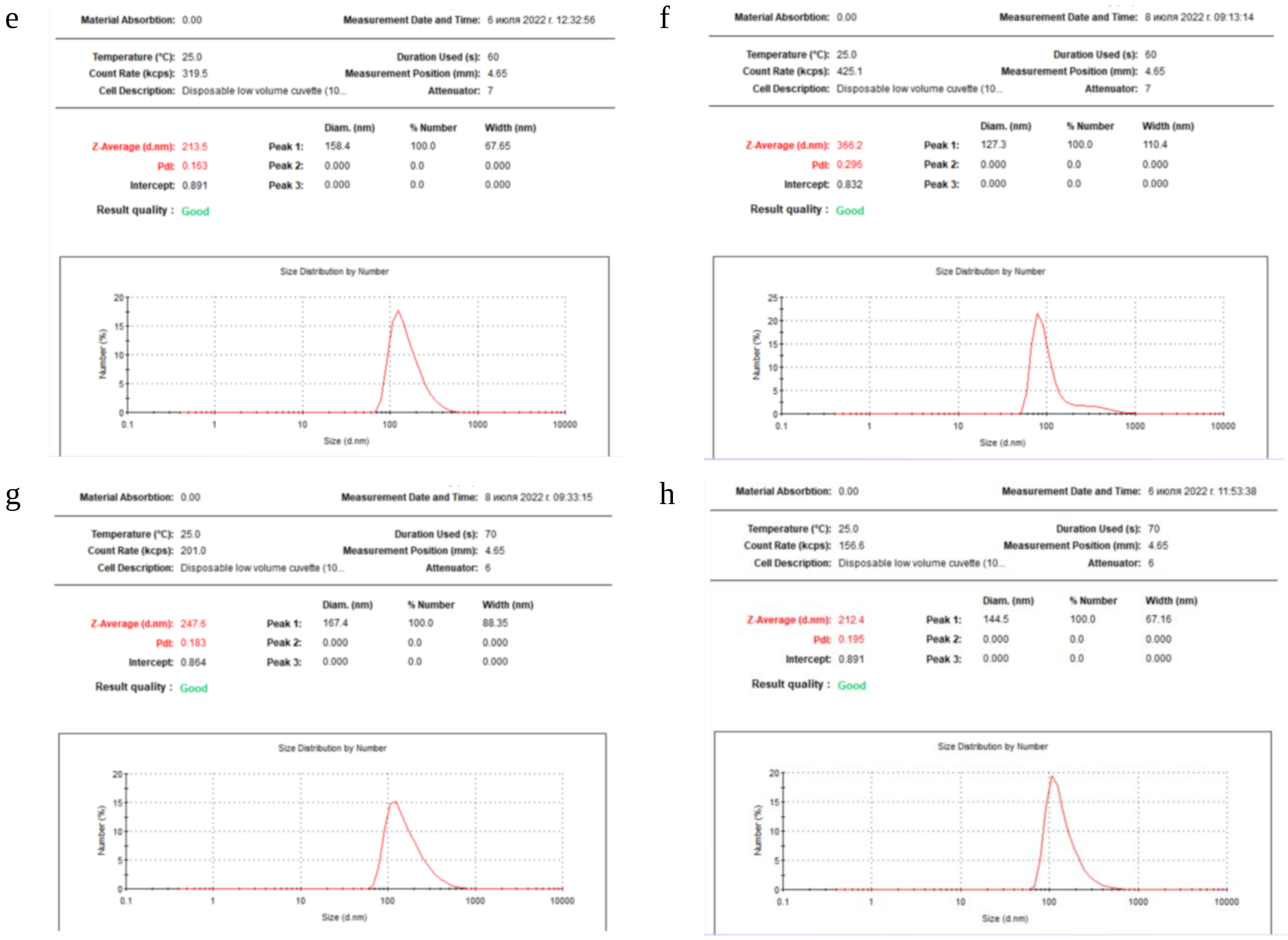

Figure S1. Particle size distribution by number in aqueous solutions of **VC10–Hal** (1:1) at concentration of 0.025 mM (a), 0.05 mM (b), 0.075 mM (c), 0.1 mM (d), 0.15 mM (e), 0.2 mM (f), 0.25 mM (g), 0.3 mM (h).

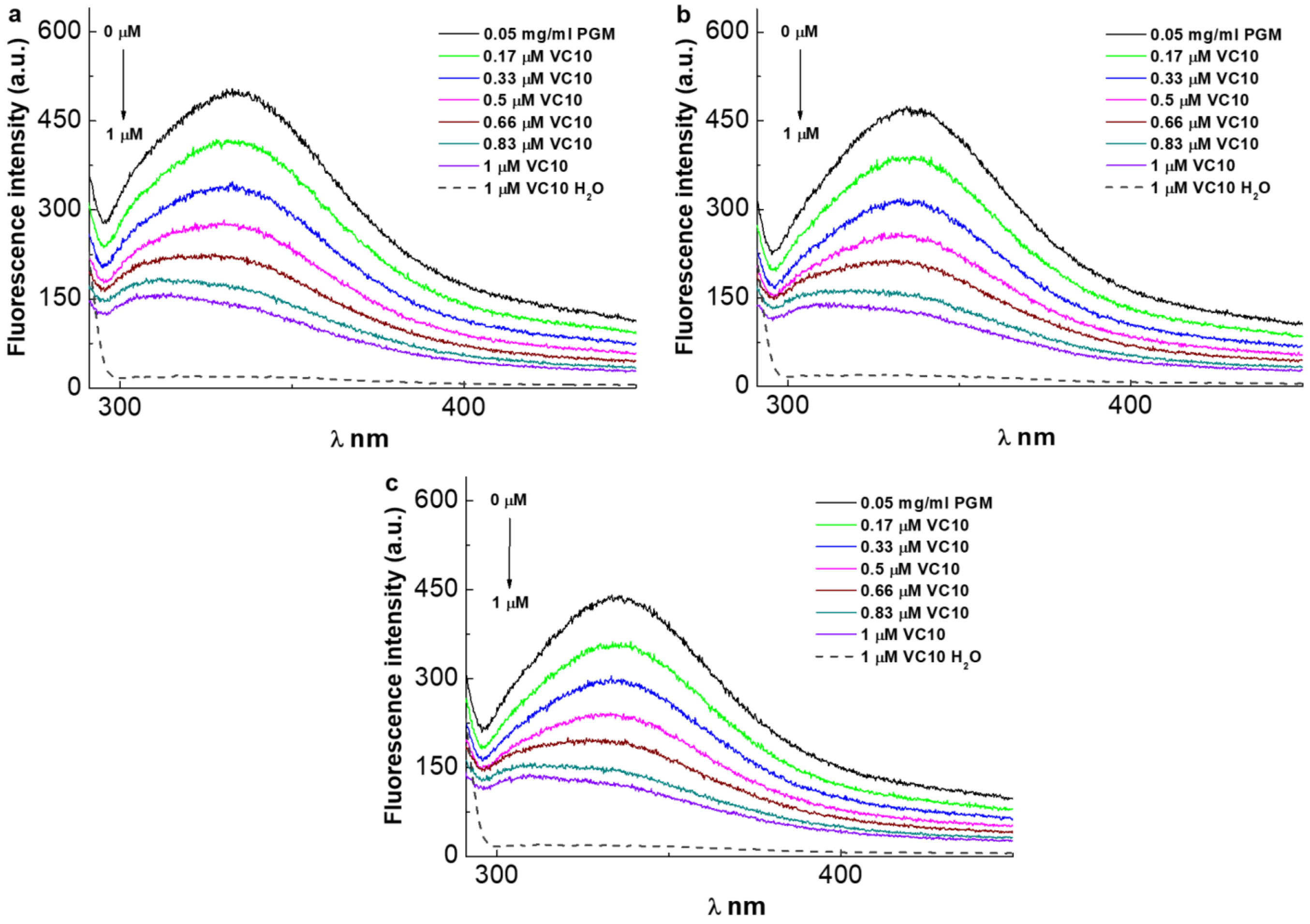

Figure S2. Steady state fluorescence spectra of PGM at a constant concentration of 0.05 mg/mL in the presence of **VC10** at 298 K (a), 304 K (b), 310 K (c).

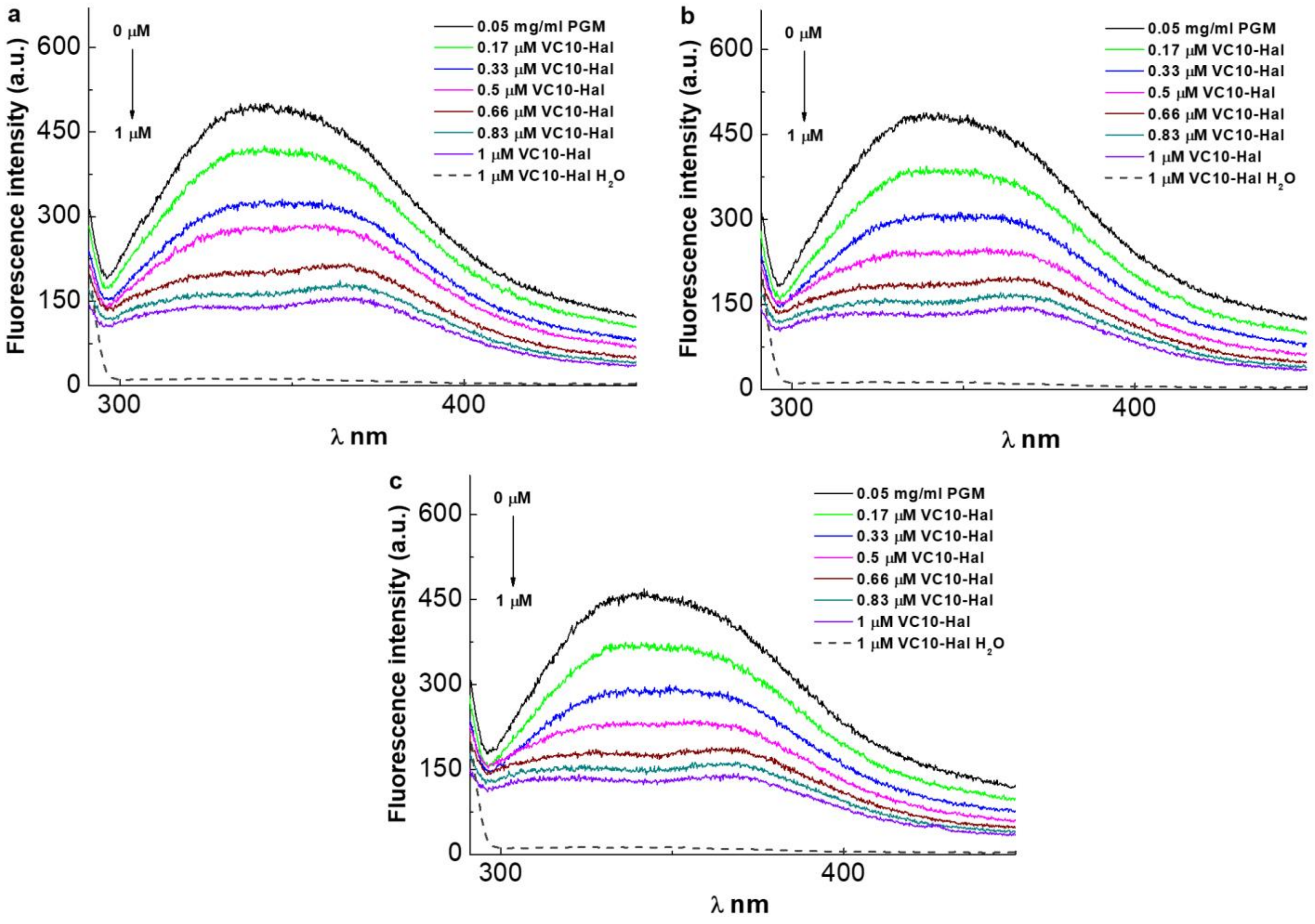

Figure S3. Steady state fluorescence spectra of PGM at a constant concentration of 0.05 mg/mL in the presence of **VC10-Hal** (1:1) at 298 K (a), 304 K (b), 310 K (c).

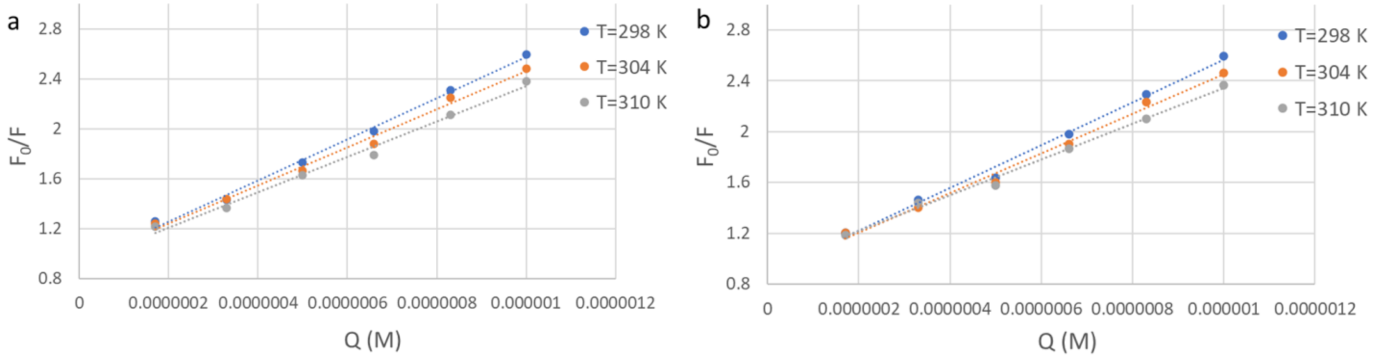

Figure S4. Stern-Volmer plots for fluorescence quenching of PGM by **VC10** (a), by **VC10-Hal** (b) at 298 K, 304 K, 310 K.

Table S1. Effect of viologen decyl calix[4]resorcinol **VC10** on the research activity of mice in the "Open field" test. (**VC10** in a dose of 1/100 of the LD_{50} and 1/50 of the LD_{50}, intraperitoneally administration, n=8).

Number of holes examined	Number of holes examined, %	Group
48,5±17,96	100±37,03	Control
19,5±0,76	40,21±1,57	**VC10**, 1/100 LD_{50}
21,83±0,91	45,02±1,88	**VC10**, 1/50 LD_{50}

Table S2. Effect of viologen decyl calix[4]resorcinol **VC10** on the motor activity of mice in the "Open field" test. (**VC10** in a dose of 1/100 of the LD_{50} and 1/50 of the LD_{50}, intraperitoneally administration, n=8).

Number of crossed lines	Number of crossed lines, %	Group
77,83±23,37	100±30,03	Control
55±6,79	70,66±8,72	**VC10**, 1/100 LD_{50}
76,83±10,17	98,72±13,06	**VC10**, 1/50 LD_{50}

Table S3. Effect of viologen decyl calix[4]resorcinol **VC10** on the the number of entrances to the center of mice in the "Open field" test. (**VC10** in a dose of 1/100 of the LD_{50} and 1/50 of the LD_{50}, intraperitoneally administration, n=8).

Number of entrances to the center	Number of entrances to the center, %	Group
3,67±1,41	100±38,35	Control
2,33±0,67	63,64±18,18	**VC10**, 1/100 LD_{50}
3,67±0,61	100±16,76	**VC10**, 1/50 LD_{50}
